# Fibrous Tumor of the Bladder Successfully Removed

**Published:** 1870-08

**Authors:** A. Reeves Jackson

**Affiliations:** No. 1026 Wabash Avenue; Chicago, Ill.


Article II.—*Fibrous Tumor of the Bladder Successfully Removed.* By A. Reeves Jackson, M.D., Chicago, Ill.

Mrs. K., a resident of Monroe county, Pennsylvania, aged 40 years; married; is the mother of four children, the youngest of whom is ten years old. She is of spare habit of body, and for a number of years has been afflicted with a large femoral hernia of the right side, for which mechanical support has been used. Her general health has usually been good.

I was called to see her December 9, 1869, and obtained the following history: During the past four or five weeks she had experienced a rather frequent desire to urinate, the passage of the water being attended with more or less pain. On several occasions the urine had been bloody. These attacks of irritability of the bladder although becoming latterly quite frequent, were not of long duration—rarely lasting more than ten or twelve hours—and had not been sufficiently severe to impel her to seek medical advice. Six days previously, (3d inst.) after one of those attacks she suddenly found herself quite unable to urinate. There was a constant painful effort to do so, accompanied with many ineffectual straining efforts. A practitioner residing in the immediate vicinity was called to see her, and a variety of powerful diuretics, purgatives, etc., were prescribed. The pain meanwhile became constant and increased in severity. In the course of two or three days the urine began to dribble away, and the patient being unable to sit up, the bedding soon became saturated. Believing that his remedies had been *too effectual* the doctor (*sic*) now commenced prescribing others, with the view of checking this profuse flow. Among these opium was given, and although it did not lessen the incontinence, it yet served a good purpose, by procuring partial relief from pain.

At the time of my visit she was still under the influence of an opiate given the previous evening. Her skin was cool and moist; tongue covered with a dry brownish coating; pulse 120, small, with a quick, sharp beat. The lower part of the abdomen was much distended, and yielded a dull sound on percussion. During the examination the patient became sufficiently aroused to recognize me, and begged that I would do something to relieve her. I introduced a catheter and drew off nearly six pints of stale, bloody, rather offensive urine, with very great relief. Suddenly the flow stopped. The enlargement of the hypogastrium although very much lessened was still distinctly perceptible, presenting the appearance of a rounded, well-defined tumor. Under the impression that some obstacle was impeding the further flow of urine, I withdrew the instrument a short distance, and this manœuvre not being successful, removed it entirely, in order to examine the openings. These being quite pervious, I was puzzled, and introduced it again. No urine appeared; and as I attempted to change the position of the instrument somewhat, I became conscious of the existence of an impediment to its usual freedom of motion. I could press it a short distance forward and toward the left side, but a strong resisting body effectually prevented the turning of the instrument, either backward or toward the right side. I passed a finger into the vagina, but could not by any manipulation succeed in feeling the catheter through its walls. A male instrument subsequently introduced gave similar results. While its point could be distinctly felt through the thin abdominal wall when pressed anteriorly, it could not be turned at all toward the right side, and very slightly toward the left; neither could its course be felt from the vagina.

This was at eight o'clock in the evening. The following morning I saw her again at an early hour and learned that she had passed a comparatively comfortable night, sleeping a considerable part of the time. There had been some dribbling of urine, and she was still unable to evacuate the bladder by any effort of her own.

Introducing a male catheter I removed a little more than a pint of bloody urine, having a strong ammoniacal odor. Again, as on the previous evening, the flow abruptly stopped; again I found that no change either in the direction or position of the instrument was sufficient to restore it; and, again, the evacuation of the bladder did not remove the persistent globular hard tumor of the abdomen. In attempting to carry the instrument about in various directions—which was done in the most gentle manner, and without producing much pain—I found the same difficulty, or rather the same impossibility, as had been noticed the evening before. On the withdrawal of the instrument, I found that its openings were partially closed by clots of fresh blood.

As the patient's residence was about sixteen miles from my own, I left a catheter with the husband, an intelligent German, instructing him sufficiently in its use, and ordered its employment at intervals of eight hours, or oftener if it should seem necessary in order to relieve pain.

I did not see my patient again for six days, at the end of which time I was summoned by telegraph, to visit her as quickly as possible. On my arrival, about noon, the husband informed me that, very early in the morning, his wife had awakened with a more intensely painful desire to urinate than she had experienced at any time since my former visit, accompanied by strong involuntary bearing-down efforts. As she could not void a single drop of urine—had not done so, in fact, since I saw her—he proceeded to introduce the catheter as usual, when, to his great surprise, he found himself prevented by the pressure of a mass of something resembling, as he said, a piece of boiled beef, having a grayish-red color, filling up and slightly protruding from the urethral orifice. In great consternation, he at once started for the nearest telegraph station, one mile distant, to summon me. On his return he discovered that the substance had disappeared into the bladder again. He now introduced the catheter without difficulty, and drew off a quantity of bloody urine, which had been set aside to await my coming, and which I found, on examination, to contain a large quantity of mucus and some pus in addition to the blood.

Several hours having elapsed since the bladder had been emptied, I introduced a catheter, and distinctly felt, as I did so, what I had not experienced on any former occasion, namely, an evident obstruction to its ready ingress. The impediment seemed to yield to moderate pressure, however, and the urine flowed freely. This latter had now a dark brown, muddy appearance, was highly offensive, and contained a large quantity of muco-pus. A small amount of fresh blood followed the withdrawal of the instrument.

With the view of making a more thorough exploration a sponge tent was introduced into the urethra and permitted to remain four hours. On removing it I was enabled to introduce the index finger sufficiently far to perceive the pressure of a tolerably dense mass with a smooth surface occupying the lower part of the bladder, and pressing against and somewhat into the vesical orifice of the urethra. A larger tent was now introduced, and as the presence of the first one had been productive of considerable pain, a full dose of Morph. Sulph. was administered at the same time. As it was impossible for me to remain with the patient, or, indeed, to see her again in less than twenty-four hours, I directed the husband to remove the tent at the end of four hours; and further, having provided him with polypus-forceps, a tenaculum, ligature-twine, etc., directed him to seize the protruding substance, should it again appear, and secure it against return until my arrival. On the following day he met me as I was approaching the house, and with a triumphant air showed me a small bottle in which was a reddish fleshy-looking mass about the thickness and nearly the length of a finger. It was tough, rather softer in some portions than others, and seemed to have been moulded to the shape of the urethra in its passage. It had made its appearance, he informed me, on the withdrawal of the tent, and he had seized it with the forceps and fingers, and making firm traction (here he had exceeded his instructions,) had caused the patient very great pain, and had broken the substance off in the urethra, where he was unable again to reach it. The urethra being now very greatly irritated and excessively painful, it was deemed advisable to suspend, for a time, operative procedures. Cloths wrung out of a hot infusion of opium and arnica flowers were applied to the genitals, and a full dose of Morphiæ given internally. She was also to have the catheter passed at regular intervals; the bowels were to be moved by enemata; and she was to have beef broth, porter etc., daily, at proper intervals.

Two days passed. All had been done according to directions. The catheter had been regularly used with variable success. Sometimes the husband was aware of the existence of an impediment to its introduction, and at others he was unable to discover anything of the kind.

The irritation of the urethra having been sufficiently lessened, I introduced as large a sponge tent as possible, and removed it at the expiration of three hours. The passage was then sufficiently dilated to permit the forefinger to enter readily into the bladder, and with its point the growth could be distinctly felt. A long narrow bladed forceps was passed in, and efforts made to grasp a portion of the tumor, with very indifferent success, however. I only succeeded in detaching small portions, and bringing them away between the blades. These attempts were attended with considerable hæmorrhage, and I finally desisted. In a short time, and while I was pondering upon the best course to adopt, the patient was seized with severe pain and vesical tenesmus, and made frantic efforts to urinate. I hastened to introduce the catheter, when I discovered a portion of the tumor showing itself at the orifice of the urethra. I at once grasped it with a pair of small placenta-forceps, and with a succession of swaying movements from side to side, drew it out about two inches. I then substituted my fingers for the instrument, and twisted the mass slowly, like a rope. The patient although suffering the most acute pain, aided its expulsion by powerful bearing-down efforts. I succeeded, in this manner, in removing a portion of the growth eight inches in length, of the thickness of the thumb. Then it gave way, and the remaining portion receded into the bladder. I was much disappointed at this termination, as I had hoped to remove the entire mass at this time. Very little bleeding attended the operation, although a few clots of blood—the result, probably, of the attempts made an hour before with the forceps—escaped by the side of the tumor, as it was being withdrawn.

During the succeeding four or five days many pieces of the now disintegrated tumor came away. They varied very greatly in size, some being quite small—mere shreds, in fact—while others were an inch or more in length. The larger pieces moulded themselves, in their passage, to the size and shape of the urethral canal. Toward the last they became exceedingly offensive, as did also the urine, which was loaded with pus, blood, and numerous shreds. The larger pieces were taken care of and placed in alcohol, at my request. The smaller ones, and that part of the growth which was softened down and passed off with the urine, of course escaped. The amount preserved weighs eight and a half ounces, and forms a mass as large as the closed fist.

The general condition of the patient now rapidly improved, so that at the end of a fortnight she was able to walk about the house. Incontinence, however, had succeeded the retention, and this still remained. The urine, which was now quite normal in quality and amount, was passing away continually, much to the disgust and discomfort of the patient. The bladder had contracted adhesions with the surrounding viscera, and its walls, thickened and hardened by inflammatory deposits, could be distinctly felt, filling a space in the hypogastric region as large as that occupied by the gravid uterus at four months. It was thus prevented from being either a retaining or an expelling organ, and I had nothing better to suggest for the patient than the use of a urinal, which was procured for her, and which she wears with a good degree of comfort.

April 30th, I saw Mrs. K. again. She had then fully regained her general health. The incontinence was improving. In certain positions of the body she could retain the urine nearly an hour, and felt hopeful that she might get entirely well. The bladder appeared also to be regaining its power; for there were times when with one or two ounces of urine retained, she found herself able to expel it with a fair degree of force.

Submitted to microscopic examination, a section of the tumor presents the same fibrous and vascular appearances as are seen in the case of uterine fibroids; and, indeed, on comparing it with a specimen of the latter, the two, both in their general character and minute structure, appear to be identical.

There are some points of interest connected with the foregoing case that seem to justify a few remarks.

It is, in the first place, as far as I am able to ascertain, the first of the kind that is recorded as having occurred to an American surgeon. Fibrous tumors are confessedly among the rarest, and perhaps are indeed the very rarest, diseases of the bladder. Thompson, in his admirable monograph on the *Urinary Organs*, says, "Thus, first, we have simple fibrous growths, springing from the walls of the bladder and wholly unassociated with the prostate; the rarest of all forms—in short, exceedingly rare, known to me personally only in museums." Gross, with his vast experience, mentions the fact that not a single case has occurred to him. This latter author states that "Zacatus Lusitanus found in the bladder of a man who died of retention of urine, a polype of the size of a goose's egg, the interior of which was occupied by a tough, glutinous substance. This was situated at the neck of the viscus, while another, a harder and more fleshy mass, existed in the body of the bladder." Baillie saw but a single case, and that one he does not particularly describe. Other cases are mentioned by Warner, Kirchner, Walther, and others. Mr. John Birkett records (*Medico-Chirurgical Transactions*, 1858,) a case which he styles one of *Fibrous Polypus of the Urinary Bladder*, but which, from the account given of the microscopical examination, was clearly not fibrous at all. Appended to the report of this latter case is a table of ten recorded cases.

In the case of Mrs. K., no one can fail to be struck with the fact that the tumor acquired so great a size in the short time over which the symptoms denoting its presence extended. Mr. Coulson makes especial mention of the fact that "they seldom acquire a large size, and their growth is not rapid." Indeed, in most of the reported cases of the disease, the history has extended over several months, and even one and two years, before the symptoms became especially marked and urgent. Here, however, we find a growth attaining the size of a large orange in a period of five or six weeks, unless, indeed, we admit that it may have existed a longer time without producing any symptoms indicative of its presence, or interfering with health.

No. 1026 Wabash avenue.

